# Smoking-induced aggravation of experimental arthritis is dependent of aryl hydrocarbon receptor activation in Th17 cells

**DOI:** 10.1186/s13075-018-1609-9

**Published:** 2018-06-08

**Authors:** Jhimmy Talbot, Raphael S. Peres, Larissa G. Pinto, Rene D. R. Oliveira, Kalil A. Lima, Paula B. Donate, Jaqueline R. Silva, Bernard Ryffel, Thiago M. Cunha, José C. Alves-Filho, Foo Y. Liew, Paulo Louzada-Junior, Fernando de Queiroz Cunha

**Affiliations:** 1 Department of Pharmacology, Ribeirao Preto Medical School, University of Sao Paulo, Av. Bandeirantes, 3900, Ribeirao Preto, SP 14049900 Brazil; 2 Department of Immunology, Ribeirao Preto Medical School, University of São Paulo, Ribeirao Preto, Brazil; 3 Division of Clinical Immunology, Ribeirao Preto Medical School, University of São Paulo, Ribeirao Preto, Brazil; 4 Université Orleans and Centre National de la Recherche Scientifique, Molecular Immunology, UMR7355, INEM, Orleans, France; 5 Institute of Infectious Disease and Molecular Medicine, UCT, Cape Town, South Africa; 6 Division of Immunology, Infection and Inflammation, Glasgow Biomedical Research Centre, University of Glasgow, Glasgow, G12 8TA UK; 7 School of Biology and Basic Medical Sciences, Soochow University, Suzhou, 215006 China

**Keywords:** Rheumatoid arthritis, Cigarette smoke, Inflammation, Polycyclic aromatic hydrocarbons, Th17

## Abstract

**Background:**

Epidemiologic studies have highlighted the association of environmental factors with the development and progression of autoimmune and chronic inflammatory diseases. Among the environmental factors, smoking has been associated with increased susceptibility and poor prognosis in rheumatoid arthritis (RA). However, the immune and molecular mechanism of smoking-induced arthritis aggravation remains unclear. The transcription factor aryl hydrocarbon receptor (AHR) regulates the generation of Th17 cells, CD4 T cells linked the development of autoimmune diseases. AHR is activated by organic compounds including polycyclic aromatic hydrocarbons (PAHs), which are environmental pollutants that are also present in cigarette smoke. In this study, we investigated the role of AHR activation in the aggravation of experiment arthritis induced by exposure to cigarette smoke.

**Methods:**

Mice were exposed to cigarette smoke during the developmental phase of antigen-induced arthritis and collagen-induced arthritis to evaluate the effects of smoking on disease development. Aggravation of articular inflammation was assessed by measuring neutrophil migration to the joints, increase in articular hyperalgesia and changes in the frequencies of Th17 cells. In vitro studies were performed to evaluate the direct effects of cigarette smoke and PAH on Th17 differentiation. We also used mice genetically deficient for AHR (*Ahr* KO) and IL-17Ra (*Il17ra* KO) to determine the in vivo mechanism of smoking-induced arthritis aggravation.

**Results:**

We found that smoking induces arthritis aggravation and increase in the frequencies of Th17 cells. The absence of IL-17 signaling (*Il17ra* KO) conferred protection to smoking-induced arthritis aggravation. Moreover, in vitro experiments showed that cigarette smoke can directly increase Th17 differentiation of T cells by inducing AHR activation. Indeed, *Ahr* KO mice were protected from cigarette smoke-induced arthritis aggravation and did not display increase in TH17 frequencies, suggesting that AHR activation is an important mechanism for cigarette smoke effects on arthritis. Finally, we demonstrate that PAHs are also able to induce arthritis aggravation.

**Conclusions:**

Our data demonstrate that the disease-exacerbating effects of cigarette smoking are AHR dependent and environmental pollutants with AHR agonist activity can induce arthritis aggravation by directly enhancing Th17 cell development.

## Background

Smoking is the major known environmental risk factor associated with the development of rheumatoid arthritis (RA) [1, 2]. Epidemiologic studies show that current and previous smoking is correlated not only with increased risk to RA development, but also to increased disease severity and reduced responsiveness to therapy [3]. Heavy smokers have twice the chance of developing RA than non-smokers [4] and an increased risk for radiographic progression [5]. Several molecular mechanisms for the increase in arthritis severity have been proposed, such as the increase in oxidative stress [6, 7], induction of enzymes that increase protein citrullination [8–11], and nicotine activation of α7-nicotinic acetylcholine receptors in neutrophils [12]. However, little is known about the molecular mechanism by which smoking affects the adaptive immune response on arthritis. Indeed, despite its pro-inflammatory effects on neutrophils [12], nicotine has an immunosuppressive effect on the immune system, suggesting that other components of cigarette smoke might be related to modulation of disease severity [13].

Among the cells for adaptive immunity, a population of interleukin 17 (IL-17)-producing CD4^+^ T cells (Th17) has been implicated as a key player in RA development and progression [14, 15]. Differentiation of Th17 cells follows a complex transcriptional regulatory network dependent on the nuclear receptor RORγt and its collaboration with other transcription factors [16]. Recent reports suggest that the transcription factor aryl hydrocarbon receptor (AHR) plays an important role in the regulation of Th17 cells development. Activation of AHR increases Th17 differentiation in vitro and *Ahr* genetic-deficient mice develop less severe collagen-induced arthritis via modulation of Th17 [17–19]. AHR, a member of the basic helix-loop-helix *Per-Arnt-Sim* (bHLH-PAS) superfamily, is a ligand-dependent transcription factor also known as ‘pollutant receptor’. This receptor is activated by a variety of organic compounds including polycyclic aromatic hydrocarbons (PAH) [20]. These are persistent organic environmental pollutants, which are present in cigarette smoke [21, 22]. We hypothesized that AHR activation by cigarette smoke components could be responsible for the aggravation of arthritis.

To understand how smoking modulates the immune response and disease aggravation, we developed a murine model of cigarette smoke exposure during antigen-induced articular disease development. Using this model, we identified that smoking-induced arthritis aggravation is dependent on AHR activation in T cells, Th17 expansion, and interleukin 17 receptor A (IL-17RA) signaling, showing a strong link between this pollutant receptor and arthritis progression. Our results suggest that AHR activation in Th17 cells might be a convergent mechanism by which different environmental pollutants aggravate autoimmune diseases.

## Methods

### Chemicals and reagents

The following materials were used: AHR agonist FICZ (6-formylindolo[3,2-b]carbazole, BIOMOL, Plymouth Meeting, PA, USA,); AHR antagonist CH223191 (1-Methyl-N-[2-methyl-4-[2-(2-methylphenyl)diazenyl]phenyl-1H-pyrazole-5-carboxamide, Tocris Bioscience, Ellisville, MO, USA); anti-CD3 and anti-CD28 (eBioscience, San Diego, CA, USA) benzo[b]fluoranthen, phorbol-12-myristate-13-acetate (PMA), ionomycin, methylated bovine serum albumin (mBSA), complete Freund’s adjuvant (CFA)with 1 mg/ml of *Mycobacterium tuberculosis* and RPMI 1640 medium (all from Sigma-Aldrich, St. Louis, MO, USA), α-IL-17a (eBioscience, anti-mouse IL-17A functional grade purified, Clone: eBioMM17F3).

### Mice

C57BL/6 wild-type (WT), DBA1/J and *Il17* receptor genetic-deficient mice on the C57BL/6 background (*Il17ra*^−/−^) [23] were bred in a specific pathogen-free animal facility at the School of Medicine of Ribeirao Preto, University of São Paulo. *Ahr*^*−/−*^ mice [24] on the C57BL/6 background and their corresponding WT mice were bred in a specific pathogen-free animal facility at the Immunologie et Neurogenetique Experimentales et Moleculaires, Orleans, France (Centre National de la Recherche Scientifique, Orleans, France). Naïve male mice (6- to 12-weeks old) were maintained in sterile, isolated, ventilated cages with controlled temperature, light conditions and ad libitum access to food and water. All the genetic-deficient mice (*Il17ra*^−/−^ and *Ahr*^*−/−*^) displayed overall good health conditions and optimal breeding. Animal husbandry and procedures were in accordance with the guidelines of the Animal Ethics Committee of the School of Medicine of Ribeirao Preto, University of São Paulo (number 038/2009).

### Induction of experimental arthritis

#### Antigen-induced arthritis (AIA)

Mice were anesthetized with *2%* isoflurane before immunization and challenge. Mice were injected subcutaneously (s.c.) on day 0 with 500 μg of mBSA in 0.2 ml of an emulsion containing 0.1 ml saline and 0.1 ml CFA, and boosted on day 7 and 14 with the same preparation in incomplete Freund’s adjuvant (IFA). Sham-immunized mice were given similar injections without mBSA. On day 21 after the first immunization, mice were challenged by intra-articular (i.a.) injection of mBSA (10 μg in 10 μl of PBS) into the right knee joint using a sterile 33-gauge syringe. Control mice were injected with 10 μl of PBS alone. Mechanical articular hyperalgesia and neutrophil infiltration were evaluated 7 h after challenge on day 21 after first immunization. For evaluation of Th17 cell frequencies draining lymph nodes (DLNs) were collected 18 days after first immunization and analyzed by flow cytometry. For histologic analysis of the knee joints, mice were challenged 7 days after first mBSA injection and euthanized 7 days later to excise the femur-tibial joints. Mice were treated with FICZ (90 μg/kg, i.p.) or vehicle [dimethyl sulfoxide (DMSO) 1% in PBS] twice (on days 12 and 17 after first immunization). We selected the in vivo dose of FICZ based in a pilot dose-response curve experiment (10, 30 and 90μg/kg) with showed that 90μg/kg induces the higher increase disease aggravation (data not shown).

#### Collagen-induced arthritis (CIA) [25]

Male DBA/1 J mice (10 weeks old) were injected intradermally (i.d.) on day 0 at the base of the tail with 200 μg of bovine type II collagen (CII, a gift of Dr. David D. Brand, University of Tennessee Health Science Center) emulsified in CFA. Mice were boosted i.d. with CII (200 μg emulsified in IFA) on day 21 and monitored daily for signs of arthritis. Scores were assigned based on erythema, swelling, or ankylosis present in each paw on a scale of 0 to 3, giving a maximum score of 12 per mouse. Mice were treated with FICZ (90 μg/kg, i.p.) or vehicle (DMSO 1% in PBS) twice (on days 12 and 17 after first immunization). All mice were euthanized on day 31 for evaluation of neutrophil infiltration in the femur-tibial joints [by myeloperoxidase (MPO) activity] and measurement of cytokines in the paws.

### Pharmacologic and immunobiologic protocols

For AHR blockage experiments mice were treated with an AHR antagonist, CH223191 (2.5 mg/kg, i.p.), every day from 11 to 19 day after first immunization. For neutralization of IL-17 activity during AIA development, mice were treated (i.p.) with an anti-mouse/rat IL-17A antibody, 5 mg/kg, daily, from 12 to 17 days after first immunization.

### Cigarette smoke exposure

Mice were exposed to smoke from commercial filter-tipped cigarettes (Marlboro 100s, Phillip Morris, yielding 0.8 mg nicotine and 10 mg tar per cigarette) in a smoking chamber modified from that of Sham [26]. Mice were exposed to one, two or three cigarettes per day (4.5 min per cigarette, five animals/chamber) twice on days 12 and 17 after first immunization. Smoke exposure was carried out by intermittently forcing air (2.5 l/min) through a burning cigarette. The intermittent cycles of forcing clean air were performed to mimic human smokers’ puff cycles and to prevent CO_2_-induced asphyxiation. Smoke cycles consisted of 15 s of active cigarette smoke followed by 30 s of forced clean air controlled by a two-way valve timer. For smoking > 1 cigarette per day, mice were rested for 3 h between each cigarette.

### Cigarette smoke-conditioned medium

The cigarette smoke-conditioned medium was prepared as follows: smoke from two filtered cigarettes (Marlboro 100s, Phillip Morris) was bubbled through 10 ml serum-free RPMI-1640 medium with a mechanical vacuum pump. The extract was filtered through a 0.22 μm filter (Millipore, Bedford, MA, USA) to remove bacteria and particles and diluted to a final volume of 400 ml with RPMI medium (five cigarettes/L). RPMI was chosen for these experiments since it has a low intrinsic AhR activity, reducing the background for the assay of AhR activation and consequently increasing the noise-to-signal ratio. Finally, CS-conditioned medium was incubated for 90 min at 37 °C, in room air. The pH of conditioned medium was not different from that of untreated RPMI. To standardize the concentration, AHR activity was evaluated by a luciferase assay. In most experiments (except described otherwise in the legends) CS-conditioned medium was used at five cigarettes per liter.

### Adoptive transfer of CD4^+^ T cells

CD4^+^ T cells (> 95% pure) were obtained from WT C57BL/6 mice by negative selection (Miltenyi Biotec, Bergisch Gladbach, Germany) and injected intravenously (5 × 10^6^ cells) into naïve *Ahr*^−/−^ mice, which were then immunized 1 day later by the AIA protocol described above.

### Determination of articular hyperalgesia

Articular hyperalgesia of the femur-tibial joint was determined as previously described [27]. In a quiet room, mice were placed in acrylic cages (12 × 10 × 17 cm high) with a wire grid floor, 15–30 min before testing for environmental adaptation. Stimulations were performed only when animals were quiet, did not display exploratory movements or defecation, and were not resting on their paws. An electronic pressure-meter consists of a hand-held force transducer fitted with a polypropylene tip (4.15 mm^2^) (IITC Inc., Life Science Instruments, Columbia, MD, USA) was used. An increasing perpendicular force was applied to the central area of the plantar surface of the hind paw to induce flexion of the femur-tibial joint followed by paw withdrawal. The pressure of force applied when the paw was withdrawn was recorded by the pressure meter. The test was performed by investigators blinded to the treatment and repeated until three consistent consecutive measurements (variation < 1 g) were obtained. Mechanical threshold is expressed in grams (g) and hyperalgesia is equated to reduction of this threshold.

### Assay for joint neutrophil infiltration

Seven hours after mBSA or PBS challenge AIA mice were euthanized and articular cavities washed twice with 10 μl PBS containing 1 mM EDTA and then diluted to a final volume of 50 μl with PBS/EDTA. Total number of leukocytes was determined in a Neubauer chamber diluted in Turk’s solution. Differential cell counts were determined in cytocentrifuged Rosenfeld stained slices (Shandon Cytospin 4, Thermo Fisher Scientific, Waltham, MA, USA) with a light microscope. Results are expressed as the number (mean ± SEM) of neutrophils per cavity.

### Cytokine measurement in CIA mice

IL-17 cytokines were measured by ELISA from homogenates of the mouse hind paws using paired antibodies (R&D Systems, Minneapolis, MN, USA).

### Determination of neutrophil accumulation in CIA

MPO activity was used as an index of neutrophil accumulation in the mouse plantar tissues and was based on a kinetic-colorimetric assay, as previously described [28].

### mRNA expression in DLNs

Inguinal DLNs were harvested 7 days after first immunization. Total RNA was extracted using TRIzol (Invitrogen, Waltham, MA, USA) following manufacturer’s instructions. Total RNA was reverse-transcribed to cDNA with oligo d(T)16 primers (Applied Biosystems, Foster City, CA, USA). cDNA was used as template for qPCR of genes of interest (SYBR green method) using ViiA7 System (Applied Biosystems). mRNA expression was analyzed using the following primers/probes (0.5 μM): *Il17*, F-CTCCAGAAGGCCCTCAGACTAC / R-GGGTCTTCATTGCGGTGG; *Gapdh*, F-GGGTGTGAACCACGAGAAAT / R-CCTTCCACAATGCCAAAGTT; *Ahr*, F-CAAATCAGAGACTGGCAGGA / R-AGAAGACCAAGGCATCTGCT; *Cyp1a1*, F-GTTCTTGGAGCTTCCCCGAT / R-CTGACACGAAGGCTGGAAGT.

### Cell sorting and in vitro Th17 cell differentiation

CD4^+^ T cells were purified from spleen and lymph nodes of C57BL/6 mice using anti-CD4 microbeads (Miltenyi Biotech) and then stained in PBS with 2% FCS for 15 min at room temperature with anti-CD4-FITC and anti-CD62L-PercP (both Biolegend, San Diego, CA, USA). The naive CD4^+^CD62L^high^ T cells were sorted using the BD FACSAria II cell sorter. After sorting, cells were activated for Th17 differentiation with anti-CD3 (2 μg/ml, plate-bound), anti-CD28 (2 μg/ml, soluble), rhTGF-β1 (2.5 ng/ml, Miltenyi Biotec), and rmIL-6 (20 ng/ml, Miltenyi Biotec). Cells were cultured for 72 h and analyzed by FACS.

### Flow cytometry and intracellular cytokine staining

Cells from DLNs or in vitro differentiated T cells were stimulated for 4 h with phorbol 12-myristate 13-aceate (PMA) (50 ng/ml), ionomycin (500 ng/ml) and a protein transport inhibitor containing monensin (BD Biosciences, Franklin Lakes, NJ, USA) before detection by staining with antibodies. Surface markers (anti-CD3 and anti-CD4) were stained in PBS with 2% FCS for 10 min at room temperature, then fixed in Cytoperm/Cytofix (BD Biosciences), permeabilized with Perm/Wash Buffer (BD Biosciences) and stained with allophycocyanin anti-mouse interferon gamma (IFN-γ) and phycoerythrin anti-IL-17A (both BD Bioscience). Cells were assayed by FACS Canto or FACS Verse (both BD Biosciences) and analyzed using Flow Jo software (Treestar, Ashland, OR, USA).

### Luciferase assay to detect AHR agonist activity

Mammalian cell line (Hepa1–6) stably transfected with a vector, pGL4.43 [luc2P/XRE/Hygro], containing a xenobiotic responsive elements (XRE) that drives transcription of the luciferase reporter geneluc2P (*Photinuspyralis*) was used. The vector contains a hygromycin resistance gene to allow selection of stably transfected cell. For the luciferase induction studies, cells were grown in 48-well plates (5 × 10^5^ cells/well) and incubated for 12 h with FICZ (300 nM), cigarette smoke-enriched medium (CSEM) or the AHR ligands benzo[b]fluoranthen. Luciferase activity in cell lysates was determined in a reaction with luciferin using a microplate luminometer. Values are presented as the mean ± SD and are expressed as relative luminescence units.

### Statistical analysis

Where appropriate (described in legends) data analysis was performed using two-way analysis of variance (ANOVA), unpaired two-tailed Student’s *t* test. Data are expressed as means ± SEM and are representative of two to three independent experiments. Means from different treatments assessed in one time point were compared by one-way ANOVA with Bonferroni’s correction. All statistical analyses were performed with the GraphPad Prism software 5.00 (GraphPad Software, San Diego, CA, USA). *P* < 0.05 was considered statistically significant.

## Results

### Smoking induces aggravation of arthritis in an IL-17RA-dependent manner

**Fig. 1:**
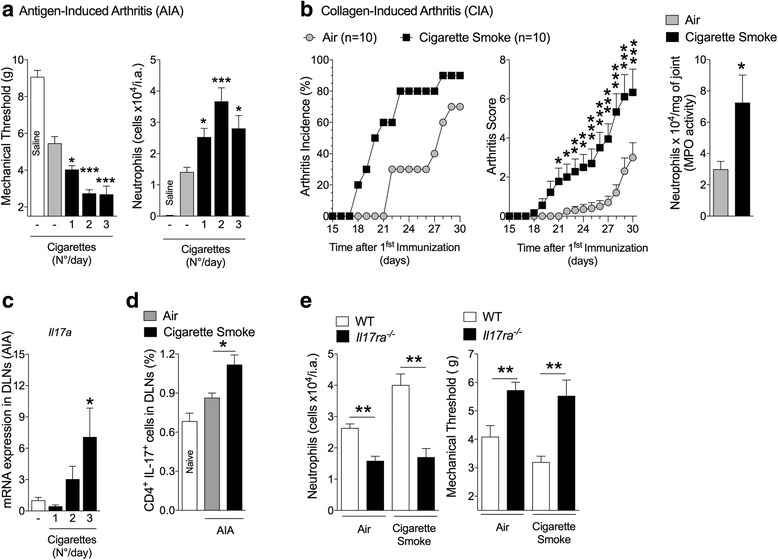
Cigarette smoke exposure induces arthritis aggravation in an IL-17RA-dependent manner. **a** AIA mice were exposed to cigarette smoke during immunization (none, one, two or three cigarettes on days 12 and 17 after immunization). Numbers of neutrophils in the joints and articular hyperalgesia was determined. **b** CIA mice were exposed to cigarette smoke (two cigarettes/day) or forced air alone (days 12 and 17 after immunization). Arthritis incidence and clinical scores were recorded up to day 30 (two-way ANOVA followed by Bonferroni post hoc test; *n* = 10, air exposed; n = 10, cigarette smoke). Numbers of neutrophils in the joint was quantified by MPO activity. Mean ± SEM, n = 10, air exposed; *n* = 9, cigarette smoke. ^*^P < 0.05, ^**^P < 0.01, ^***^P < 0.001 (two-sided Student’s *t* test). **c** *Il17a* mRNA expression in DLNs of AIA mice was determined 19 days after the first immunization (as in b). Mean ± SEM, *n* = 5/group. ^*^P < 0.05, ^**^P < 0.001*,* one-way ANOVA followed by Bonferroni post hoc test. **d** Th17 frequency in DLNs of AIA mice. Mean ± SEM, *n* = 4/group, ^*^P < 0.05, one-way ANOVA followed by Bonferroni post hoc test. **e** AIA in WT or *Il17ra*^−/−^ mice exposed to cigarette smoke or air (as in b). Mean ± SEM, *n* = 5/group, ^***^P < 0.001, one-way-ANOVA followed by Bonferroni post hoc test. All data are representative of two independent experiments. *DLN* draining lymph nodes, *IL-17* interleukin 17, *MPO* myeloperoxidase

We developed a murine model of smoking exposure during arthritis development to evaluate the mechanisms by which environment pollutants might induce aggravation of autoimmune diseases. C57BL/6 mice were exposed to cigarette smoke during the immunization phase of AIA. Exposure to cigarette smoke induced a dose-dependent increase in mechanical articular hyperalgesia and neutrophil infiltration into the joints (Fig. 1a). In the model of CIA, exposure to cigarette smoke also exacerbated disease incidence, arthritis scores and neutrophil infiltration into the joints (Fig. 1b). We observed a cigarette dose-dependent increase in the expression of *Il17* mRNA in the DLNs of mice during AIA development (Fig. 1c). The frequencies of Th17 cells were also higher in the DLNs of mice exposed to cigarette smoke than control mice (Fig. 1d). Importantly, smoking-induced arthritis aggravation, determined by neutrophil infiltration in the joints and mechanical articular hyperalgesia in the AIA model, was absent in mice genetically deficient of IL-17RA (*Il17ra*^−/−^) (Fig. 1e). These results indicate that cigarette smoking-induced aggravation of articular inflammation is associated with an increase of Th17 frequencies and dependent on IL-17RA.

### Smoking-induced arthritis aggravation is dependent on AhR activation

**Fig. 2:**
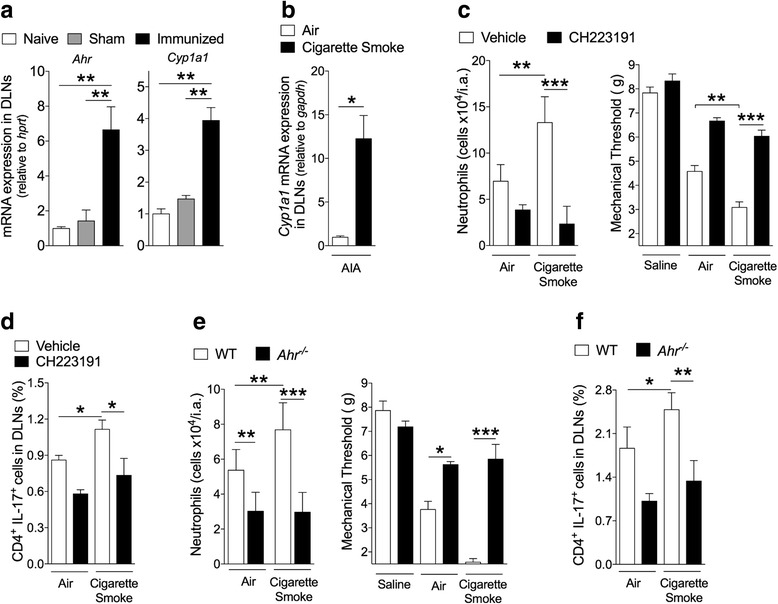
Smoking-induced arthritis aggravation and increase in Th17 activity are AHR-dependent. **a** *Ahr* and *Cyp1a1* mRNA expression in DLNs from non-immunized (Naïve), sham-immunized (Sham) and Immunized mice (AIA) 7 days after first immunization. Mean ± SEM, n = 4/group, ^**^P < 0.01 by one-way ANOVA followed by Bonferroni post hoc test. **b** *Cyp1a1* mRNA expression in DLNs of AIA mice 6 h after exposure to cigarette smoke (two cigarettes, day 12 after immunization). Mean ± SEM, n = 4 mice/group. ^*^P < 0.05, one-way ANOVA followed by Bonferroni post hoc test. **c** AIA mice were exposed to cigarette smoke and treated with CH223191. Number of neutrophils in the joints and articular hyperalgesia were determined. Mean ± SEM, n = 4/group, ^**^P < 0.01*,* ^***^P < 0.001, one-way ANOVA followed by Bonferroni post hoc est. **d** Th17 frequency in DLNs of AIA mice exposed to cigarette smoke and treated with CH22319. Mean ± SEM, n = 4/group, ^*^P < 0.05, one-way ANOVA followed by Bonferroni post hoc test. **e** *Ahr*^*−/−*^ or WT mice during AIA were exposed to cigarette smoke. Mean ± SEM, n = 4/group. ^*^P < 0.05, ^**^P < 0.01, ^***^P < 0.001, one-way ANOVA followed by Bonferroni post hoc test. **f** Frequency of Th17 cell in the DLNs of WT and *Ahr*^*−/−*^ mice exposed to cigarette smoke. Mean ± SEM, n = 4/group. ^*^P < 0.05, ^**^P < 0.01, one-way ANOVA followed by Bonferroni post hoc test. Data are representative of at least two independent experiments. *CH223191* 1-Methyl-N-[2-methyl-4-[2-(2-methylphenyl)diazenyl]phenyl-1H-pyrazole-5-carboxamide, *DLN* draining lymph nodes, *IL-17* interleukin 17

We then investigated the mechanism by which smoking induces the increase in Th17 development. We observed an increased expression of *Ahr* and *Cyp1a1* (downstream of *Ahr* activation) in the inguinal DLNs of mice during AIA (Fig. 2a). Moreover, elevated levels of *Cyp1a1* mRNA were clearly evident in the DLNs of mice under AIA when exposed to cigarette smoke compared to those mice exposed to air alone (Fig. 2b). Since AHR is a key inducer of Th17 and *Cyp1a1* is a downstream transcription factor of *Ahr* these results indicate that smoke exposure is inducing AHR activation during Th17 generation in vivo.

Consistent with this notion, smoking-induced arthritis aggravation and increase in Th17 frequencies in AIA was markedly inhibited when the mice were treated with an AHR antagonist, CH223191 (Fig. 2c and d). Importantly, while WT mice developed exacerbated hyperalgesia, increased neutrophil infiltration and Th17 frequencies when exposed to cigarette smoke during AIA, this aggravation was completely absent in *Ahr*^*−/−*^ mice (Fig. 2e and f). These results therefore demonstrate that smoking-induced arthritis aggravation is dependent on AHR activation and elevation of Th17.

### Cigarette smoke and polycyclic aromatic hydrocarbons have direct effects on Th17 differentiation and arthritis aggravation

**Fig. 3:**
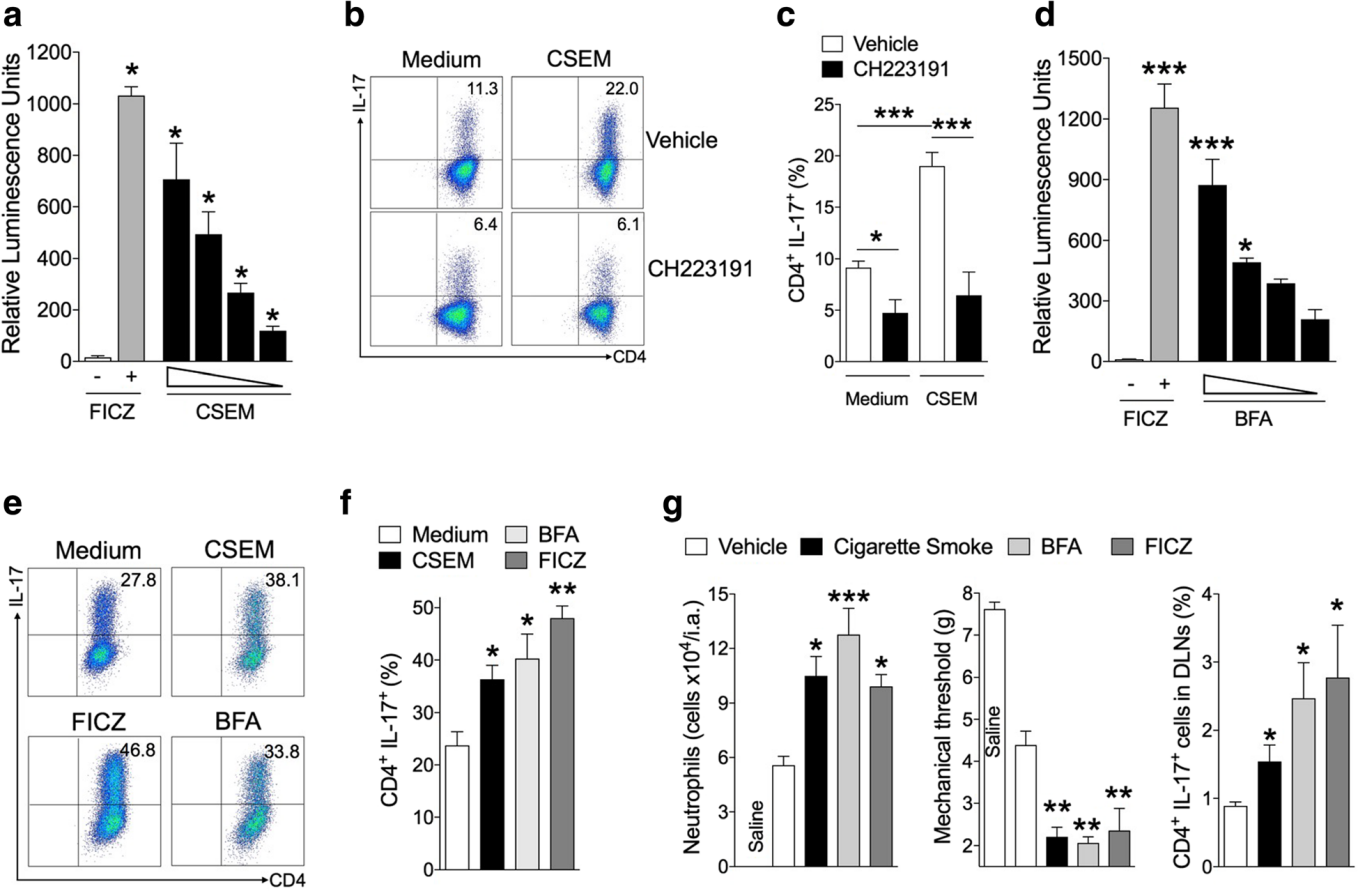
Cigarette smoke and polycyclic aromatic hydrocarbons affect Th17 differentiation and arthritis aggravation. **a** RLU (relative luminescence unit) in Hepa1–6.pGL4.43 cells induced by FICZ (300 nM) or cigarette smoke-enriched medium (CSEM: 5, 0.5, 0.05, 0.005 cigarettes/L). Mean ± SEM, *n* = 3, ^*^P < 0.05, one-way ANOVA followed by Bonferroni post hoc test. **b** and **c** Naive CD4^+^ T cells from C57BL/6 mice were polarized under Th17 conditions under control medium or CSEM (five cigarettes/L) ± AHR antagonist (CH223191, 30 μM). **(d)** RLU in Hepa1–6.pGL4.43 cells induced by FICZ (200 nM) or benzo(b)fluoranthene (BFA: 200, 20, 2 and 0.2 nM). Data are mean ± SEM, n = 3, ^*^P < 0.05 and ^***^P < 0.001 by one-way ANOVA followed by Bonferroni post hoc test. **e** and **f** Naive CD4^+^ T cells were polarized under Th17 conditions in the presence of BFA (200 nM), FICZ (200 nM) or CSEM (five cigarettes/L). Mean ± SEM, n = 3, ^*^P < 0.05, ^**^P < 0.01, one-way ANOVA followed by Bonferroni post hoc test. **g** AIA mice were exposed to cigarette smoke (two cigarettes) or treated with BFA (100 μg/kg) or FICZ (90 μg/kg). Number of neutrophils in the joints, articular hyperalgesia and Th17 cells in DLNs. Mean ± SEM, *n* = 5/group, ^*^P < 0.05, ^**^P < 0.01, ^***^P < 0.001, one-way ANOVA followed by Bonferroni post hoc test. Data are representative of two to three independent experiments. *BFA* benzo[b]fluoranthen, *CH223191* 1-Methyl-N-[2-methyl-4-[2-(2-methylphenyl)diazenyl]phenyl-1H-pyrazole-5-carboxamide, *CSEM* cigarette smoke-enriched medium, *DLN* draining lymph nodes, *FICZ* 6-formylindolo[3,2-b]carbazole, *IL-17* interleukin 17

Using a reporter cell line for AHR ligands (Hepa1–6 *pGL4.43*), we observed that CSEM induces a concentration-dependent activation of AHR (Fig. 3a). This CSEM was able to increase in vitro differentiation of Th17 cells (Fig. 3b and c), which was abolished by the AHR antagonist CH223191 (Fig. 3b and c). These data suggest a strong link between direct influences of AHR activation in Th17 cells.

We then investigated whether PAH present in cigarette smoke could modulate Th17 differentiation and exacerbate arthritis. As a PAH prototype we used benzo[b]fluoranthen (BFA), which is found in high concentrations in cigarette smoke [29] and exhibits a potent AHR agonist activity (Fig. 3d). BFA induced an increase in Th17 differentiation in vitro to a similar level as that induced by the AhR endogenous agonist, FICZ, or CSEM (Fig. 3e and f). In vivo, BFA and FICZ also induced arthritis aggravation and an increase of the frequency of Th17 in the draining lymph nodes of AIA mice (Fig. 3g).

Together, these data demonstrate that environmental pollutants with an AHR agonist activity can increase of Th17 cell development and mediate diseases aggravation.

### AHR activation in CD4 T cells induces arthritis aggravation and is IL-17A-dependent

**Fig. 4:**
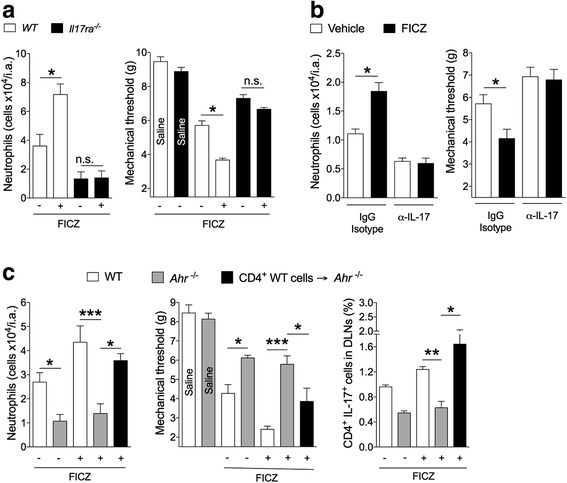
AHR activation in T cells induces arthritis aggravation in an IL-17RA-dependent manner. **a** AIA was induced in *WT* or *Il17ra*^−/−^ mice and treated with vehicle or FICZ (90 μg/kg) during immunization. Neutrophil in the joints and hyperalgesia were determined 7 h after challenge. Mean ± SEM, n = 5/group, ^*^P < 0.05; *n.s,* not significant, one-way ANOVA followed by Bonferroni post hoc test. **b** AIA was induced in WT mice treated with anti-IL-17 (α-IL-17) or the isotype control antibody at the same time as the mice were treated with vehicle or FICZ. Neutrophil in the joints and hyperalgesia were determined 7 h after challenge. Mean ± SEM, n = 5/group, ^*^P < 0.05; *n.s,* not significant, one-way ANOVA followed by Bonferroni post hoc test. **c** AIA was induced in *WT* or *Ahr*^*−/−*^ mice. The mice were treated or not with FICZ. One group of *Ahr*^−/−^ mice (CD4^+^*WT* cells → *Ahr*^*−/−*^) received CD4^+^ T cells from *WT* mice 1 day before the first immunization. Data are mean ± SEM, n = 5/group, ^*^P < 0.05, ^**^P < 0.01, ^***^P < 0.001, one-way ANOVA followed by Bonferroni post hoc test. All data are representative of two independent experiments. *DLN* draining lymph nodes, *FICZ* 6-formylindolo[3,2-b]carbazole, *IL-17* interleukin 17

Similar to what was observed for AIA mice exposed to cigarette smoke (Fig. 1e), the AHR agonist FICZ failed to induce arthritis aggravation of AIA in *Il17ra*^*−/−*^ mice (Fig. 4a). Furthermore, the disease-exacerbating effect of FICZ was also abolished by the treatment with a neutralizing anti-IL-17 antibody (Fig. 4b). To investigate whether the disease-exacerbating effects of AHR activation on increased articular inflammation was CD4^+^ T cell-dependent, we performed AIA in chimeric mice. Purified WT CD4^+^ T cells were adoptively transferred into *Ahr*^*−/−*^ mice 1 day before first immunization. *Ahr*^*−/−*^ mice were not responsive to FICZ in terms of neutrophil infiltration in the joints, articular hyperalgesia, nor increase in Th17 cell frequency in the DLNs (Fig. 4c). In contrast, these responses were completely restored by the adoptive transfer of CD4^+^ T cells from WT mice (Fig. 4c). These results therefore indicate that a direct activation on CD4^+^ T cells via Th17 development in vivo is sufficient for the AHR-dependent aggravation of articular inflammatory disease.

## Discussion

Data presented here demonstrate that cigarette smoking-induced aggravation of arthritis is dependent on the activation of AHR and the subsequent induction of Th17 and production of IL-17A. Our study therefore provides a hitherto unrecognized direct linear link among the key players: cigarette smoking and polycyclic aromatic hydrocarbon, AHR activation, IL-17A production, and articular inflammation. The transcriptional mechanism by which AhR regulates Th17 cell differentiation and expansion is not fully understood. Since forced expression of AhR did not impact on *Rorc* expression in Th17 cells [18] other mechanisms might be involved in the regulation of Th17 development. Analysis of the *Il17* loci revealed a putative binding site for AhR on an E-box element at the *Il17* promoter, suggesting that AhR might directly regulate IL-17 expression [30]. Moreover, AHR might also regulate Th17 cell proliferation by the transcriptional control of *miR-132/212* clusters, which are important for Th17 development [31].

In humans, the activation of AhR in Th17 cells might play a distinct and additive pro-inflammatory role for RA aggravation (e.g. free radicals induction of oxidative stress, induction of peptidyl arginine deiminases) [6–12]. Moreover, although other cell types (e.g. ILC3 and γδ T cells) are also sources of IL-17 during inflammatory process [32], our data from adoptive transfer experiments suggest that CD4 T cells are the main target responsible for arthritis aggravation induced by AhR activation. This is consistent with an earlier report showing that AHR in CD4 T cells, but not in other immune cells, is important for arthritis development and Th17 expansion [19]. Our study not only corroborates the previous findings of an AhR cell autonomous role in CD4 T cells for arthritis development, but identify that the major environmental risk for RA development, cigarette smoking, induces disease aggravation through an AhR-dependent increase in Th17 cell response.

It is worth mentioning that several putative AhR agonists have been described to have immunosuppressive activity (e.g. TCDD, Tetrandine, Simomenine) [17, 33–36]. Some of these ligands, including the dioxin 2,3,7,8-Tetrachlorodibenzodioxin (TCDD), have been described as being able to increase Th17 differentiation [37] as well as increase the generation of regulatory T cells [17, 37]. In our experiments, the role of AhR on smoking-induced arthritis aggravation is demonstrated by the lack of smoking effects on mice genetically deficient to AhR. Moreover, the AhR antagonist CH223191 also reduced smoking-induced arthritis aggravation, suggesting blockage of this receptor could be an important target for RA management. The genetic evidence describing that AhR plays a major role on Th17 differentiation [18, 37], and the AhR-independent activity of dioxins [38], suggest that some of the immunosuppressive effects observed could be dependent of unknown biologic targets of AhR ligands.

In this study, we also identified that PAHs can also induce arthritis aggravation by modulation of Th17 generation in vivo and in vitro. It would be of considerable importance to analyze the active PAHs of cigarette smoke associated with the exacerbation of RA as several AHR ligands have been identified in cigarette smoke [39–41]. Moreover, PAHs and other putative AhR agonists might not only increase AhR activity by direct binding to this receptor, but also by inhibition of oxidases that degrade the endogenous ligands of AhR [42], which increase the group of compounds that could affect the described AhR-Th17 relationship.

Given the promiscuous nature of AHR binding, other environmental risk factors linked to RA by epidemiologic evidence are likely to be sources of AHR ligands and could be related to disease development [21, 41, 43–46]. One example is the intriguing association of living proximity to heavy traffic, exposure to traffic pollution, and RA development [47]. In our experiments, a brief exposure to cigarette smoke and AhR activation can induce aggravation of arthritis, while in humans the timeline for duration of cigarette smoke exposure and RA development might be longer. However, epidemiologic evidence suggests that even low exposure during the lifetime to cigarette smoking increases the risk for RA [48]. In humans, several factors account for the break of self-tolerance and RA development (e.g. specific microbiota, genetic background). In our proposed model, cigarette smoke exposure might potentiate the events that lead to disease development and aggravation.

## Conclusions

This study shows that smoking-induced arthritis aggravation is dependent on AhR activation in Th17 cells and IL-17Ra signaling. Given the pervasive pathogenic nature of Th17 cells, our findings may also extend to other diseases such as inflammatory bowel disease [49–51], atherosclerosis [52, 53], cancer [54, 55] and periodontitis [56, 57] all of which have a strong link to smoking.

## Abbreviations

AHR: Aryl hydrocarbon receptor
AIA: Antigen-induced arthritis
BFA: Benzo[b]fluoranthen
CFA: Complete Freund’s adjuvant
CH223191: 1-Methyl-N-[2-methyl-4-[2-(2-methylphenyl)diazenyl]phenyl-1H-pyrazole-5-carboxamide
CIA: Collagen-induced arthritis
CSEM: Cigarette smoke-enriched medium
DLN: Draining lymph nodes
DMSO: Dimethyl sulfoxide
FICZ: 6-formylindolo[3,2-b]carbazole
IFA: Incomplete Freund’s adjuvant
IL-17: Interleukin 17
IL-17Ra: Interleukin 17 receptor A
mBSA: Methylated bovine serum albumin
MPO: Myeloperoxidase
PAH: Polycyclic aromatic hydrocarbons
PBS: Phosphate-buffered saline
PMA: Phorbol-12-myristate-13-acetate
RA: Rheumatoid arthritis
